# Combined effect of bisphosphonate and recombinant human bone morphogenetic protein 2 on bone healing of rat calvarial defects

**DOI:** 10.1186/s40902-015-0015-3

**Published:** 2015-07-02

**Authors:** Ho-Chul Kim, Jae-Min Song, Chang-Joo Kim, Sang-Yong Yoon, In-Ryoung Kim, Bong-Soo Park, Sang-Hun Shin

**Affiliations:** 1 Department of Oral and Maxillofacial Surgery, School of Dentistry, Pusan National University, 626-787 Yangsan, Mulgeum-eup Korea; 2 Department of Oral Anatomy and Cell Biology, School of Dentistry, Pusan National University, Yangsan, Korea; 3 Biomedical Research Institute of Pusan National University, Yangsan, Korea; 4 Department of Oral and Maxillofacial Surgery, Good Gang-An Hospital, Busan, Korea

**Keywords:** Bisphophonate, Bone morphogenetic protein 2, Bone regeneration, Histology, Alendronate

## Abstract

**Background:**

This study aimed to investigate new bone formation using recombinant human bone morphogenetic protein 2 (rhBMP-2) and locally applied bisphosphonate in rat calvarial defects.

**Methods:**

Thirty-six rats were studied. Two circular 5 mm diameter bony defect were formed in the calvaria using a trephine bur. The bony defect were grafted with Bio-Oss® only (group 1, n = 9), Bio-Oss® wetted with rhBMP-2 (group 2, n = 9), Bio-Oss® wetted with rhBMP-2 and 1 mM alendronate (group 3, n = 9) and Bio-Oss® wetted with rhBMP-2 and 10 mM alendronate (group 4, n = 9). In each group, three animals were euthanized at 2, 4 and 8 weeks after surgery, respectively. The specimens were then analyzed by histology, histomorphometry and immunohistochemistry analysis.

**Results:**

There were significant decrease of bone formation area (p < 0.05) between group 4 and group 2, 3. Group 3 showed increase of new bone formation compared to group 2. In immunohistochemistry, collagen type I and osteoprotegerin (OPG) didn’t show any difference. However, receptor activator of nuclear factor κB ligand (RANKL) decreased with time dependent except group 4.

**Conclusion:**

Low concentration bisphosphonate and rhBMP-2 have synergic effect on bone regeneration and this is result from the decreased activity of RANKL of osteoblast.

## Background

In oral and maxillofacial field, restoring bony defect and enhancing bone regeneration is one of the most interesting subjects. Grafting bone materials such as autogenous, allogenic, and heterogenous bone are most widely used to restore bony defect. There have been studied to improve the efficiency of the bone regeneration. In recent times, there are reports of studies using growth factors (BMP, PDGF, TGF-β, IGF etc.) to enhance the efficiency of such bone grafting [1].

Bone morphogenetic protein (BMP) is one of the TGF-β super family, known to form new bones and cartilage. It is reported that BMP −2, −4, −5, −6, −7 etc. have been revealed to have osseoinductibility [2–4]. Out of these, rhBMP-2 which is obtained from recombinant DNA technology from mammalian cells is known to stimulate differentiation of osteoblast in cell experiments and to induce osteogenesis in animal experiments [5–7].

Bisphosphonate is widely used in the treatment of osteoporosis and osseous metastasis of cancer, and it is known to play a role of reducing bone loss by reducing or stopping the function of osteoclast [8, 9]. As increased use of bisphosphonate, there have been reported bisphosphonate related osteonecrosis of jaw (BRONJ) due to suppressed osteoclastogenesis [10, 11]. On the other hand, there are studies regarding low concentration of locally applied bisphosphonate accelerates the healing of the tooth extraction socket, bone fracture or increase the bone density of the interface after titanium implant placement [12–14]. We hypothesized that rhBMP-2 and low concentration bisphosphonate have synergic effect for bone regeneration. The aim of this study was to investigate the combined effect of rhBMP-2 and locally applied bisphosphonate of bone regeneration in a rat model.

## Methods

### Preparation of animal

Thirty-six Sprague-Dawley male rats (15 weeks old, Koatech, INC. Korea) weighing between 250 g and 300 g comprised the animal experimental model used. The animals were housed individually in standard rat cages maintained under an ambient temperature of 24 °C to 26 °C and a 12/12 h light/dark cycle. The animals had free access to drinking water and standard laboratory pellets. This study was conducted at the Pusan National University Institutional Animal Care and Use Committee (PNU-2011-000254 ).

### Experimental materials and surgical procedures

Each bony defect was stuffed by Bio-Oss®, inorganic bovine bone, as xenograft material. rhBMP-2 was produced in E. coli using genetic engineering (Cowellmedi Co, Busan, Korea). As for rhBMP-2, 100 μg/1 mL concentrations were used. Alendronate (Sigma, St. Louis, MO, USA) was used as bisphosphonate. 1 mM (low concentration) and 10 mM (high concentration) alendronate were conducted this study.

The animals were anesthetized with a mixture of 10 mg/kg of xylazine hydrochloride (Rumpun® Bayer, Korea) and 100 mg/kg of ketamine chloride (Ketalar®, Yuhan Corporation, Korea). The dorsal area of the rat cranium was shaved before surgery, and the surgical field was prepared with an iodine solution. A midline skin incision was performed on the skull, and the periosteum with the temporalis muscle was reflected laterally. Two symmetrical round 5 mm diameter bony defects were then formed in the calvaria using 5 mm diameter trephine (Hee Sung Corp., Seoul, Korea). The bony defect were grafted with Bio-Oss® only (group 1, n = 9), Bio-Oss® wetted with rhBMP-2 (group 2, n = 9), Bio-Oss® wetted with rhBMP-2 and 1 mM alendronate (group 3, n = 9) and Bio-Oss® wetted with rhBMP-2 and 10 mM alendronate (group 4, n = 9). Then, the muscle layer was closed with 4–0 Vicryl® sutures in a continuous fashion, and the skin with 3-0 Vicryl^Ⓡ^ sutures. Gentamycin at 5 mg/kg was injected for prevention of infection after surgery. The animals from each group were sacrificed at 2, 4 and 8 weeks after surgery. The skin was dissected, the calvaria harvested and immediately immersed in a 10 % tempered solution of formaldehyde.

### Histology

Each specimen was fixed in 10 % formaldehyde solution, decalcified in formic acid for 48 h, and embedded in paraffin. Serial cross-sections (5 μm) were cut through the larger diameter of the defect and stained with hematoxylin-eosin (H-E). The H-E stains reveal the cellular reactions indicating bone formation. The slides were photographed with the use of a virtual slide system (Scanscope CS system, Aperio Technologies, Vista,CA).

### Histomorphometric analysis

The Aperio Technologies Scanscope CS system is useful for calculating new bone formation areas on H-E stained slides. The calculation, involving just the drawing of the newly formed bone outlines, is easily done. Slides in each group were scanned by virtual slide system microscopy (X100), and then 2 slides from each group were selected in the 2, 4, 8 weeks. To calculate the new bone formation area, 4 sites were randomly selected for each slide, the photographs of which were 0.600 mm × 0.500 mm. In this study, we applied 2 statistical methods to the significance testing of each group. The dependent variables of the control and experimental groups were averages and standard deviations. The difference of the dependent variables in each group for the 2, 4, 8 weeks was analyzed by Kruskal-Wallis test and Tukey’s post hoc test. The collected data were analyzed with the use of SPSS 18.0 software (SPSS Inc., Chicago, IL, USA).

### Immunohistochemical analysis

The 5 μm thick slice maintained at 60 °C in an oven for an hour. After that, the sample was washed with distilled water after being hydrated using several steps of alcohol after the paraffin was removed. This treatment was repeated four times for five minutes with xylene. And then the sample was washed three times with the buffer solution for three minutes. In order to remove the intrinsic peroxide within the tissue, the sample was reacted with 0.3 % H_2_O_2_ solution. The sample was washed four times with buffer solution in order to inhibit the unusual combination within the tissue, after reacting with blocking serum (goat ImmunoCruz staining system, Santa Cruz Biotechnology, Inc., USA) for an hour, and reacting for a night with a dilution of each primary antibody, OPG, RANKL, Collagen type I (Santa Cruz biotechnology, OPG 1:120, RANKL 1:40, collagen type I 1:100). After being washed with the buffer solution, the sample was reacted with a secondary antibody labeled Biotin for an hour, washed four times again with the buffer solution, and reacted with the enzyme conjugate streptavidin (goat immunocruz staining system, Santa Cruz Biotechnology) for an hour at room temperature.

The tissue section was again washed in PBS three times for 10 min and was colored for 3 min in a solution mixed with diaminobenzidine chromogen and hydrogen peroxidase. The section was then washed in Tris buffer, PBS and distilled water for 10 min each. After that, it was counterstained with Harris hematoxylin and then mounted.

After each staining we evaluated the slides by immunohistochemical reactivity for RANKL using a scoring system of −, +, ++, and +++, which corresponded to absent staining, weak staining (<25 % of cells), moderate staining (<50 % of cells), and strong staining (>50 %), respectively. In the evaulation of OPG, collagen Type I expression, the boundary of cell counting was using 50 % and 75 % instead of 25 % and 50 %.

## Results

### Histologic finding

#### H-E stain

**Fig. 1:**
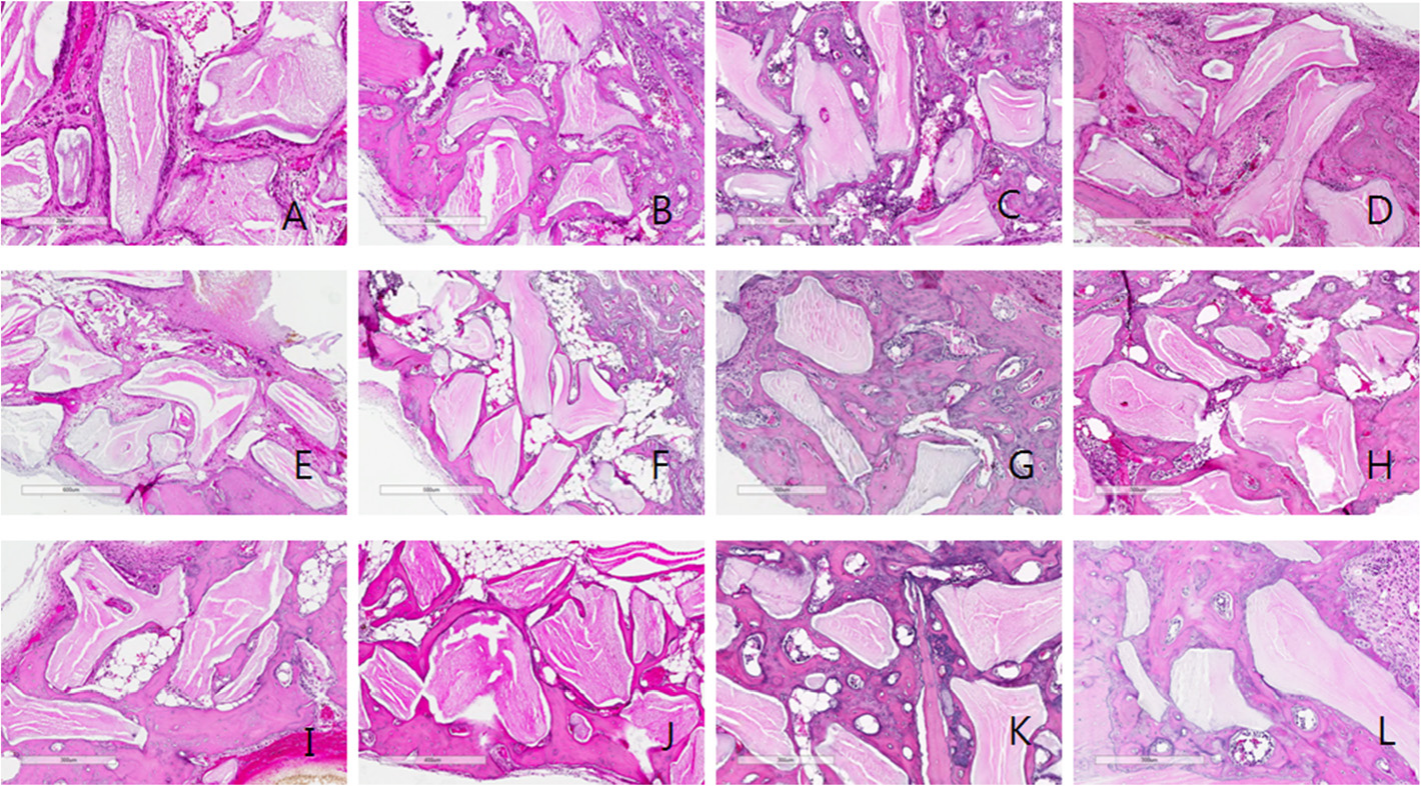
Histological view at 2, 4 and 8 weeks. **a** 2 week group 1; **b** 2week group 2; **c** 2week group 3; **d** 2 week group 4; **e** 4 week group 1; **f** 4 week group 2; **g** 4 week group 3; **h** 4 week group 4; **i** 8 week group 1; **j** 8 week group 2; **k** 8 week group 3; **l** 8 week group 4. (H-E stain, Magnification X 100)

After grafting, at 2 week collagen fiber arrangement was finding in all group. Osteoblast proliferation was observed that occurred in early stage of new bone formation . At 4 week, relatively small bone formation was observed at group 2, 3, 4. At 8 week, all group showed the aspect of complete mineralization (Fig. 1).

#### Degrees of inflammatory reaction

**Fig. 2:**
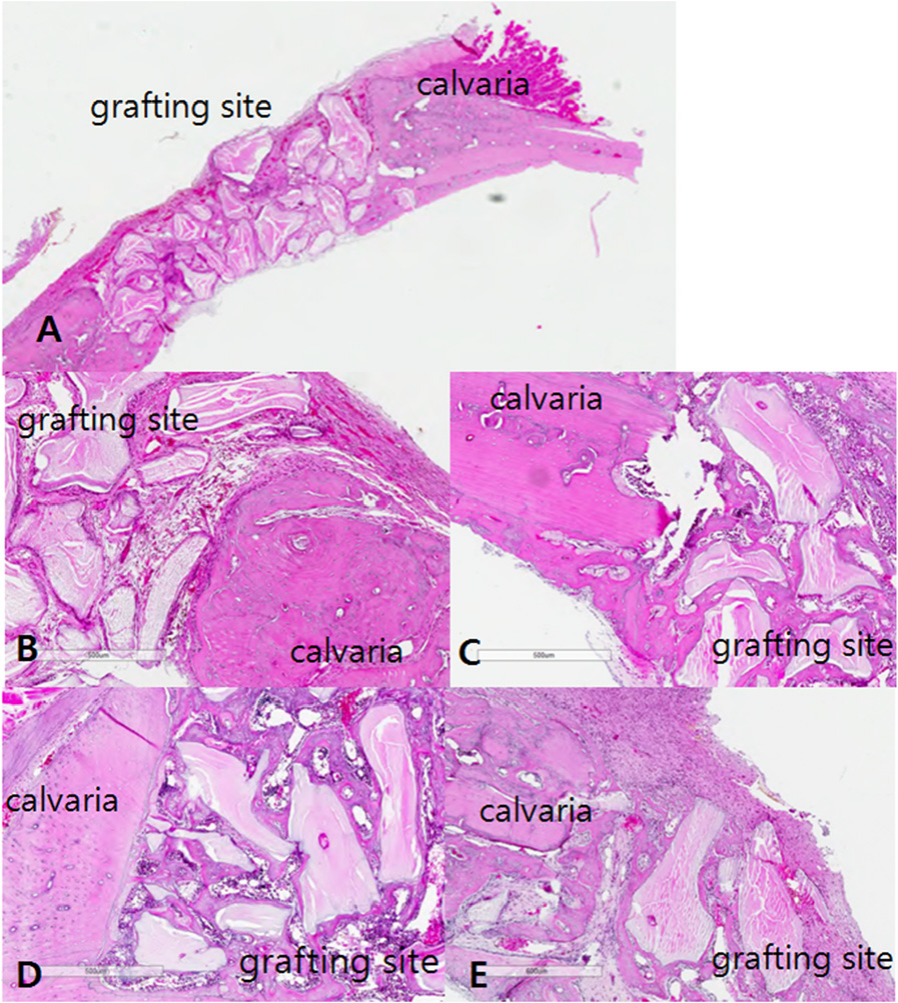
Inflammatory reaction of grafted site at 2 week. **a** Gross specimen; **b** Group 1; **c** Group 2; **d** group 3; **e** Group 4. In group 4, inflammatory cells and capillaries were observed. (H-E stain, Magnification X 100)

Relative inflammatory reaction intensity was evaluated for each groups. We examined the specimens at 2 week in each group because most of healing process and inflammatory phase are almost finished within 2 week. In group 1, 2, 3, any remarkable inflammatory reaction was not observed, but mild inflammation was observed in group 4 (Fig. 2, Table 1).

**Table 1:** Inflammatory reaction in each group

Time	Group 1	Group 2	Group 3	Group 4
2 week	±	±	±	+

(±; minimal, +; mild)

### Histomorphometric analysis

**Fig. 3:**
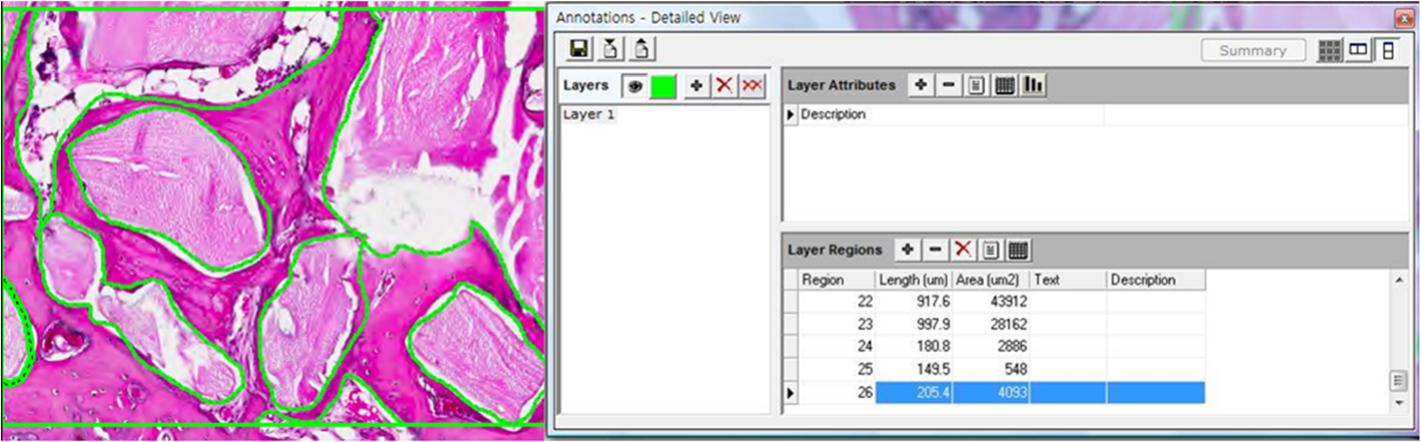
Measurement of bone formation area by Aperio imaging scope. New bone formation can be calculated just drawing the outlines of newly formed bone. H-E stain, Magnification X 100

After measuring the area where bones were formed by randomly designating 4 locations from one specimen, an average was calculated and shown by ratio. The width of the designated rectangle was set to 0.3 mm [2] (Fig. 3).

In 2 weeks, mean extent of bone formation was 10.46 % in group 1, 17.63 % in group 2, 19.73 % in group 3 and 12.41 % in group 4. In 4 weeks, that was 18.79 % in group 1, 23.21 % in group 2, 28.49 % in group 3 and 21.27 % in group 4. In 8 weeks, the extent was 31.93 % in group 1, 34.55 % in group 2, 38.98 % in group 3 and 30.68 % in group 4.

The bone formation area was calculated from bone formation ratio and expressed as mean ± standard deviation. In 2, 4 week, there exist significant difference among each groups. In 8 week there were not any difference among groups. In 2 week, group 2 and 3 showed increased bone formation significantly compared to group 1. And group 3 increased larger than group 4 significantly. In 4 week, group 3 showed increased bone formation area compred to group 1 and 4 (Tables 2 and 3).

**Table 2:** Measurement of bone formation area

Time	Group 1	Group 2	Group 3	Group 4	*P* value
2 week	0.031 ± 0.006	0.052 ± 0.004	0.059 ± 0.008	0.037 ± 0.006	0.002^*^
4 week	0.056 ± 0.004	0.069 ± 0.01	0.085 ± 0.006	0.063 ± 0.008	0.004^*^
8 week	0.096 ± 0.010	0.104 ± 0.013	0.117 ± 0.01	0.092 ± 0.009	0.072

(Unit ; mm^2^, *p < 0.05)

**Table 3:** Post hoc test results (Tukey HSD)

		*P* value	
Groups		2 week	4 week
Group 1	Group 2	0.014^*^	0.182
	Group 3	0.003^*^	0.003^*^
	Group 4	0.681	0.661
Group 2	Group 3	0.636	0.067
	Group 4	0.065	0.765
Group 3	Group 4	0.012^*^	0.020^*^

(*P < 0.05)

### Immunohistochemistric finding

#### Collagen type I

**Fig. 4:**
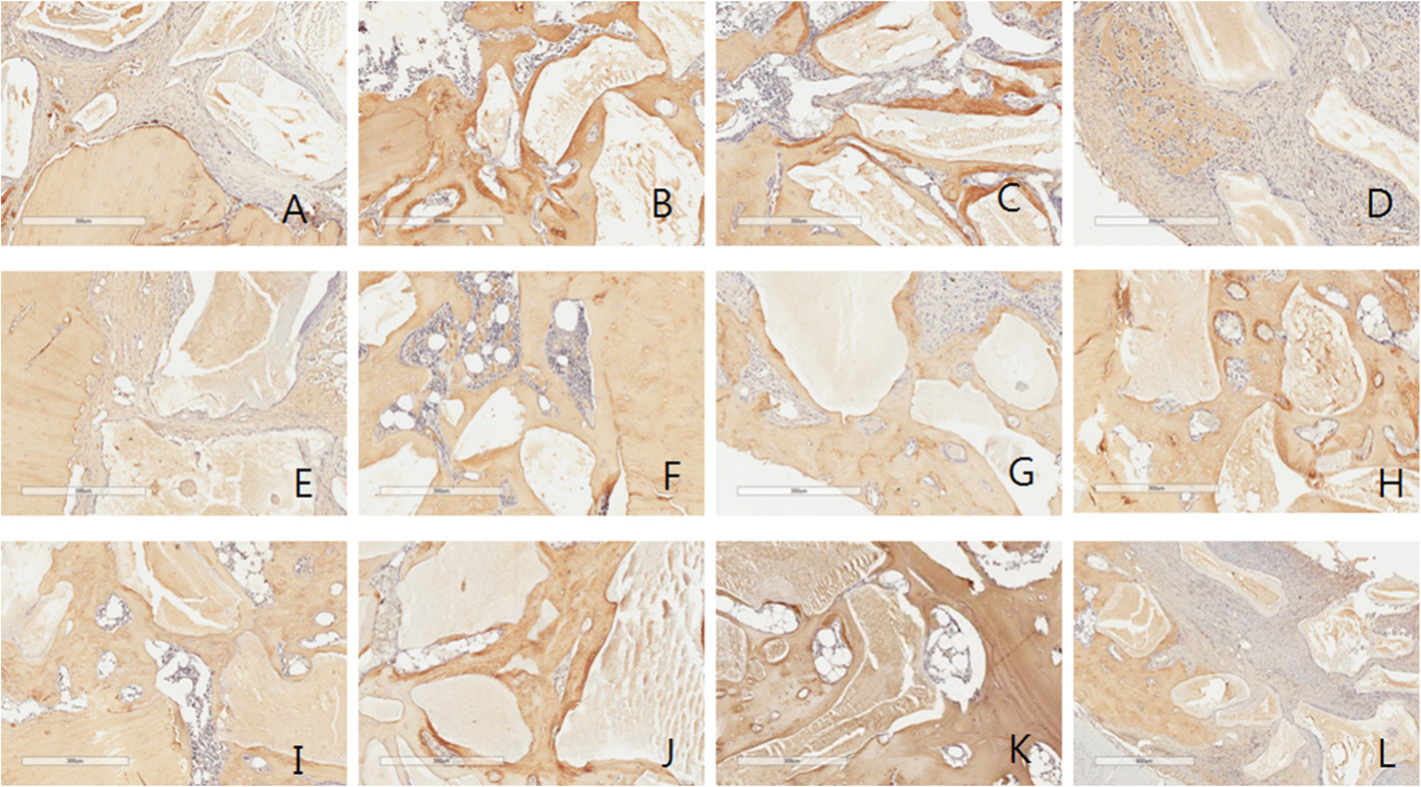
Collagen type I antibody reaction of grafted sites. **a** 2 week group 1; **b** 2week group 2; **c** 2week group 3; **d** 2 week group 4; **e** 4 week group 1; **f** 4 week group 2; **g** 4 week group 3; **h** 4 week group 4; **i** 8 week group 1; **j** 8 week group 2; **k** 8 week group 3; **l** 8 week group 4. Magnification X 100

With regard to collagen, high expression was examined in group 1,2,3. There was no difference among three groups, but group 4 shows less expression of collagen type I relatively than other group (Fig. 4, Table 4).

**Table 4:** Immunohistochemistry analysis of collagen type I, OPG and RANKL

Antibody	Time/week	Groups			
		Group 1	Group 2	Group 3	Group 4
Collagen type I	2	++	+++	+++	+
	4	++	++	++	++
	8	+++	+++	+++	+
	2	++	++	++	+
OPG	4	+/++	++	+/++	+
	8	++	++	+++	+
	2	++	++	+	++
RANKL	4	++	+++	−/+	++
	8	+	+	+	++

(−; no immunoreactivity, +; weak immunoactivity, ++; moderate immunoactivity, +++; strong immunoactivity, Abbreviations: *OPG* osteoprotegerin, *RANKL* receptor activator nuclear factor κB ligand)

#### OPG

**Fig. 5:**
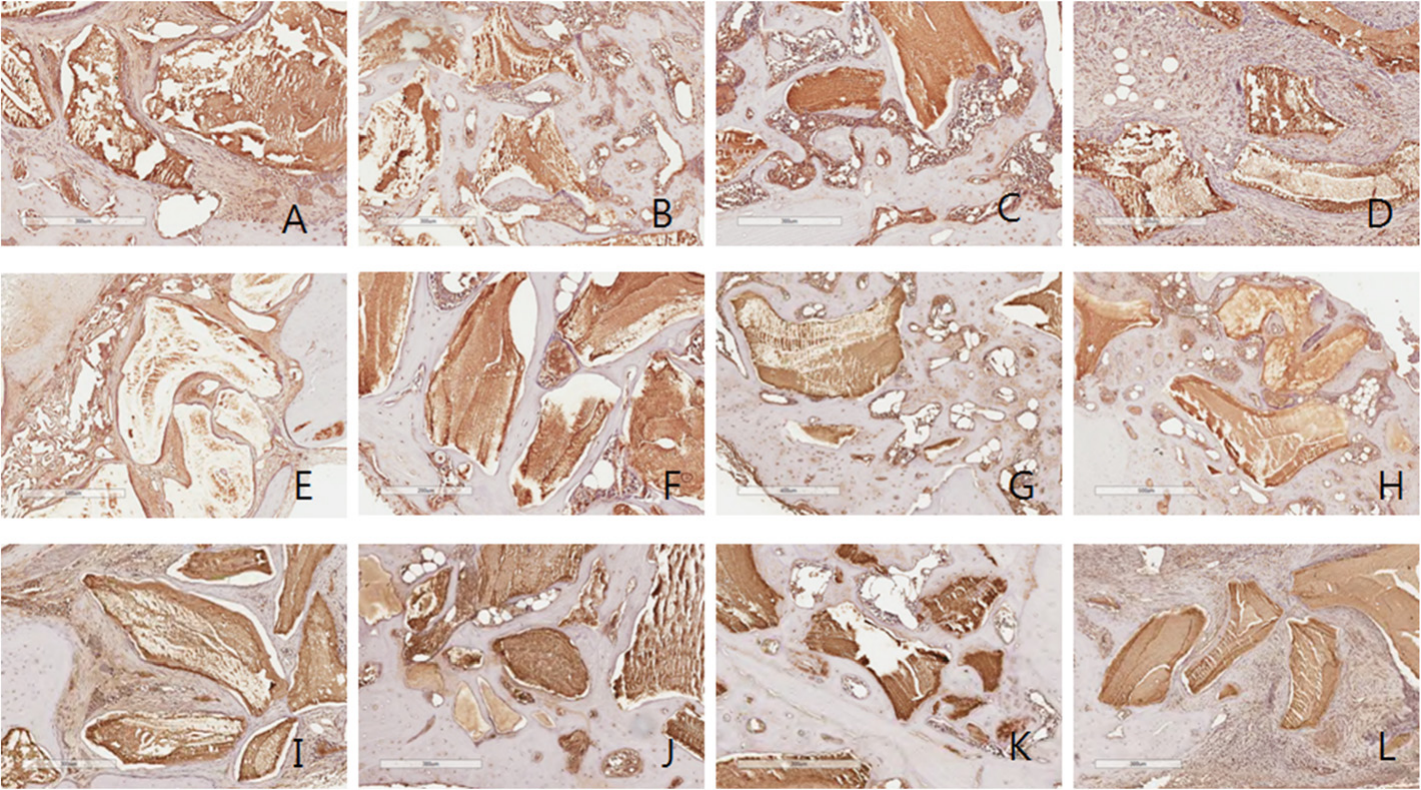
OPG antibody reaction of grafted sites. **a** 2 week group 1; **b** 2week group 2; **c** 2week group 3; **d** 2 week group 4; **e** 4 week group 1; **f** 4 week group 2; **g** 4 week group 3; **h** 4 week group 4; **i** 8 week group 1; **j** 8 week group 2; **k** 8 week group 3; **l** 8 week group 4. Magnification X 100

In the analysis of OPG expression, it shows moderate expression at 2, 4 weeks, and decrease at 8 weeks. Like collagen type I, no differeces were examed among group 1,2,3. In group 4, OPG did not manifest well (Fig. 5, Table 4).

#### RANKL

**Fig. 6:**
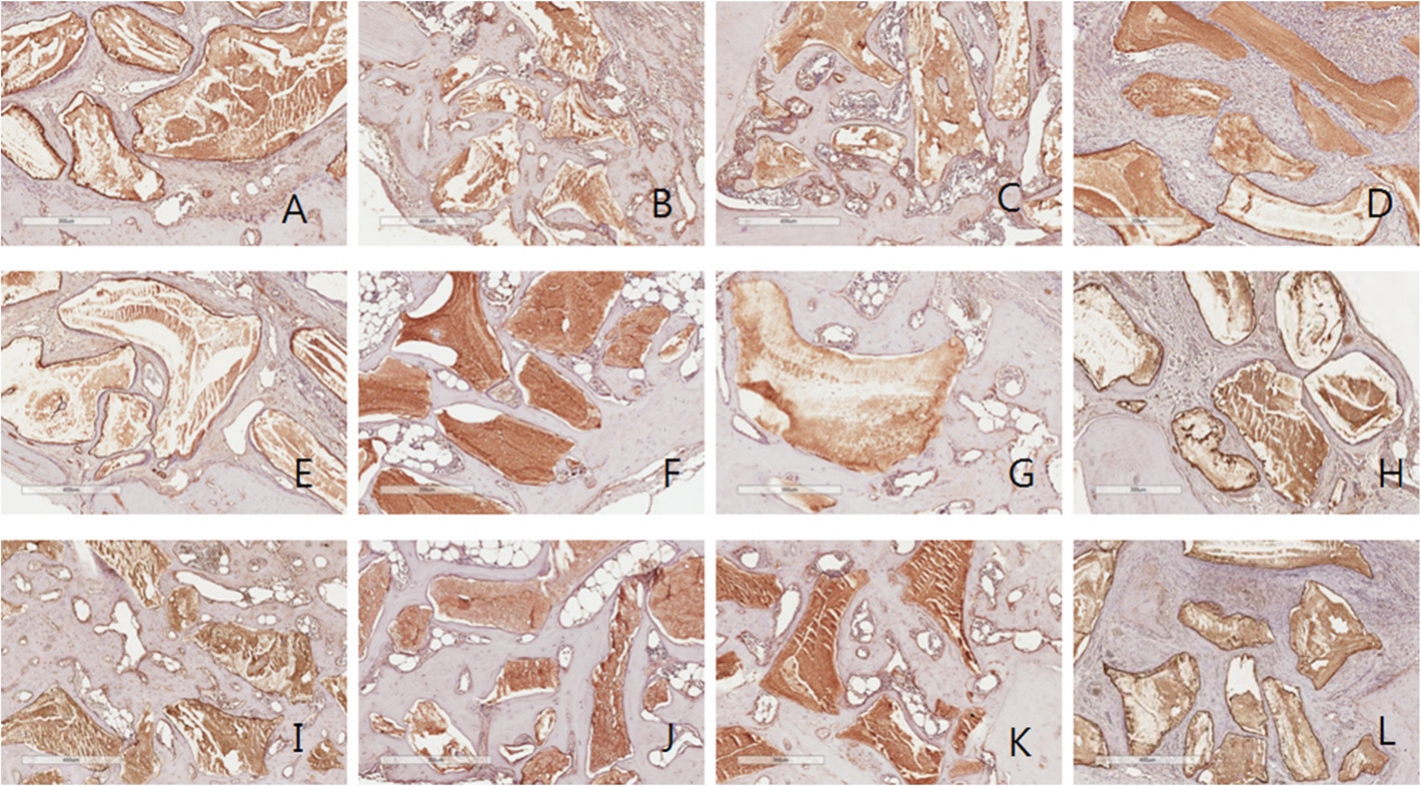
RANKL antibody reaction of grafted sites. **a** 2 week group 1; **b** 2week group 2; **c** 2week group 3; **d** 2 week group 4; **e** 4 week group 1; **f** 4 week group 2; **g** 4 week group 3; **h** 4 week group 4; **i** 8 week group 1; **j** 8 week group 2; **k** 8 week group 3; **l** 8 week group 4. Magnification X 100

In the case of RANKL, expression of RANKL was decreased with time dependant except group 4. Manifestation seems to be slightly reduced in the 2 and 4 week stages in group 3. It continues to be manifested moderate in group 4 (Fig. 6, Table 4).

## Discussion

In this study we aimed to investigate if rhBMP-2 and locally applied bisphosphonate have synergic effect on bone regeneration. Since Urist reported the osteoconductive potential of BMP 2, numerous studies were conducted on rhBMP-2 [2]. Active studies are being carried out after it was approved for use in dental field by the FDA (INFUSE® Bone Graft, Medtronic Spinal and Biologics, Memphis, Tenn) in 2007. BMP-2 have an effect on precursor cells like mesenchymal stem cells which differentiating into osteoblast. It is widely known that BMP, when local applied, accelerates bone regeneration [15].

Phamacologic mechanism of alendronate is inhibition of farnesyl diphosphate synthase in the mevalonate pathway essential for the prenylation of proteins in osteoclasts. This causes mechanical inhibition of osteoclast adhesion on the bone margin and osteoclast apoptosis [16]. The effect of BPs on osteoclasts could also be produced by osteoblasts. Osteoblast/stromal cells regulate osteoclastogenesis by M-CSF, RANKL and OPG [17, 18]. Through cell-to-cell contact of osteoblast/stromal cells with osteoclasts, RANKL and M-CSF induce osteoclast progenitor cells to differentiate into osteoclasts. There are reports that low concentration bisphosphonate stimulate the proliferation and differentiation of osteoblast [19–22]. Also systemic or local short period application of bisphosphonate improves bone regeneration due to pharmacological effects in vivo [23–26].

In the histomorphometry analysis, in the 2 week, significant difference was observed in group 2 and 3 compared to the group 1 and 4. In spite of group 2 was much bone formation, there was not significant difference between group 2 and 3. In the results of the 4 week, group 3 was only significant difference compare to other group and highest bone formation. It seems ordinary BMP was involved in the early stage of osteogenesis and BMP was main role in bone formation on 2 week. In 4 and 8 weeks there was more bone formation in group 3. It seems alendronate was main role in suppression of osteoclastic activity at initiate of bone remodeling with BMP.

RANKL is known as osteoclast differentiation factor and it is known to accelerate and stimulate their differentiation [27]. The manifestation of RANKL appears on the surface of marrow stromal cells, immature osteoblasts or mesenchymal cells [28]. OPG is an important regulator of osteoclastogenesis via it’s binding to RANKL of the effects of RANKL. OPG is role of inhibiting bone resorption. When its concentration is reduced, the resorption processes may prevail and bone loss occurs [29, 30]. In the immunohistochemistry findings, in the case of collagen, most came out uniformly without a significant difference in most of the specimen, but because osteogenesis occurred relatively not well in group 4, it seems collagen manifestation also did not appear well. Likewise, even in the case of OPG, it seems to be not manifested well because of a relatively weak of osteogenesis in the high concentration group. In the case of RANKL, it seems there is a slight reduction in manifestation from the group 3 in the early phase, and this seems to show a suppression of the osteoclastic activity of alendronate. In group 4, RANKL continues to be moderate manifested, this is seems to be relatively less bone formatinon and suppression of osteoclastic activity a weak of osteogenesis, though its mechanism is vague.

Unlike previous experiments, this experiment was designed in combination with elements that can be easily applied to preclinical practice. First, as scaffolds or carrier, it has used deproteinized bovine bone, and was not applied by adsorbing alendronate for a long period of time, and direct used 1 mM, 10 mM together with rhBMP-2. And this experiment has attempted to perform application by using BMP and bisphosphonate together. The concentration of BMP was determined from the manufacturer, and the concentration of bisphosphonate is known to use a concentration of 1 mg/ml in many theses, but it was difficult to find grounds for these. Therefore, we used the concentration of 1 mM/ml (0.325 mg/ml) that was suggested purpose of prevent root resorption in the delayed replantation of an extracted tooth as a standard [31–33]. In group 3, there were statistically significant differences in 2 and 4 week compared to group 1 and 4. It seems there is a great bone forming effect since it showed a significant difference in the 4th week compared to group 2. While it is difficult to provide an accurate interpretation of its mechanism since these are not results from substituting one condition but from using compositive methods, the group using a combination of a rhBMP-2 and 1 mM of alendronate seems to have faster and better bone formation than the control group.

## Conclusion

This study was conducted to investigate whether alendronate and rhBMP-2 have combined effect on bone regeneration. Our results indicate that low concentration bisphosphonate and rhBMP-2 have synergic effect on bone regeneration and this is result from the decreased activity of RANKL of osteoblast. Though further studies are needed to find out an appropriate concentration, it seems capable of being clinically applied in bony defect.

## Abbreviations

BMP: Bone morphogenetic protein
rhBMP-2: Recombinant human bone morphogenetic protein 2
BP: Bisphophonate
BRONJ: Bisphosphonate related osteonecrosis of jaw
H-E: Hematoxylin-eosin
OPG: Osteoprotegerin
RANKL: Receptor activator of nuclear factor κB ligand
M-CSF: Macrophage colony-stimulating factor
